# Metastatic lymph node ratio as an important prognostic factor in advanced gallbladder carcinoma with at least 6 lymph nodes retrieved

**DOI:** 10.1007/s00423-023-03119-5

**Published:** 2023-09-28

**Authors:** Junke Wang, Fei Liu, Wenjie Ma, Haijie Hu, Fuyu Li

**Affiliations:** Division of Biliary Tract Surgery, Department of General Surgery, West China Hospital, Sichuan University, Chengdu, 610041 Sichuan China

**Keywords:** Gallbladder carcinoma, Metastatic lymph node ratio, Prognostic factor

## Abstract

**Background:**

The metastatic lymph node (LN) ratio (LNR) has shown to be an important prognostic factor in various gastrointestinal malignancies. Nevertheless, the prognostic significance of LNR in gallbladder carcinoma (GBC) remains to be determined.

**Methods:**

From January 2007 to January 2018, 144 advanced GBC patients (T2–4 stages) who underwent curative surgery with at least 6 LNs retrieved were enrolled. Receiver operating characteristic (ROC) curve was performed to identify the optimal cut-off value for LNR. The clinicopathological features stratified by LNR level were analyzed. Meanwhile, univariate and multivariate Cox regression proportional hazard models were performed to identify risk factors for overall survival (OS).

**Results:**

The optimal cut-off point for LNR was 0.28 according to the ROC curve. LNR>0.28 was associated with higher rate of D2 LN dissection (*P*=0.004) and higher tumor stages (*P*<0.001). Extent of liver resection, extrahepatic bile duct resection, tumor stage, LNR, margin status, tumor differentiation, and perineural invasion were associated with OS in univariate analysis (all *P*<0.05). GBC patients with LNR≤0.28 had a significantly longer median OS compared to those with LNR>0.28 (27.5 vs 18 months, *P*=0.004). Multivariate analysis indicated that tumor stage (T2 vs T3/T4; hazard ratio (HR) 1.596; 95% confidence interval (CI) 1.195–2.132), LNR (≤0.28 vs >0.28; HR 0.666; 95% CI 0.463–0.958), margin status (R0 vs R1; HR 1.828; 95% CI 1.148–2.910), and tumor differentiation (poorly vs well/moderately; HR 0.670; 95% CI 0.589–0.892) were independent prognostic factors for GBC (all *P*<0.05).

**Conclusions:**

LNR is correlated to advanced GBC prognosis and is a potential prognostic factor for advanced GBC with at least 6 LNs retrieved.

## Introduction

Gallbladder carcinoma (GBC) is relatively unusual, but the most prevalent malignancy in the biliary tract system [1]. Due to nonspecific clinical manifestations, most GBC patients are diagnosed at advanced stages with a poor prognosis [2]. The 5-year overall survival (OS) of GBC was reported less than 10% [3, 4]. Even so, radical resection offers the only hope to achieve long-term survival. For Tis or T1 GBC, simple cholecystectomy is an adequate procedure, and no lymph node (LN) dissection is needed. However, for advanced GBC with T2 or higher stages, radical cholecystectomy including liver resection and LN dissection is essential to achieve an R0 resection [5]. Besides, postoperative evaluation of prognostic factors is crucial for predicting prognosis in GBC patients.

LNs status is one of the most valuable prognostic factors after radical resection [6]. The incidence of LN metastases in GBC varies by tumor stages, up to 45–80% in T2–4 stages [6, 7]. Previous studies have indicated that GBC patients without LN metastases had significantly better prognosis than those with LN metastases [8]. However, many issues about LN metastases in GBC still await clarification including the required extent of LN dissection and the accurate stratification of prognosis [6, 9]. Although the 8th edition of the American Joint Committee on Cancer (AJCC) classification, which defines LN staging based on the number of metastatic LNs, is the most accepted schema for GBC patients [10], scholars still explored alternative LN staging schemes including the metastatic lymph node ratio (LNR).

Prognostic significance of the LNR, that is the ratio of the number of positive LNs to the number of LNs retrieved, has been confirmed in various gastrointestinal malignancies, including gastric cancer and pancreatic cancer [11–13], but not yet for GBC. Several investigators have reported that LNR as an independent prognostic factor in curative resected GBC [14, 15], whereas others argue that metastatic LNs rather than LNR was the optimal LN staging system in evaluating GBC prognosis. Significantly, these studies have ignored the discrepancy of the number of LNs retrieved. The 8th edition of the AJCC tumor staging system for GBC recommended that at least 6 LNs should be retrieved [10]. However, there is a large discrepancy in the number of retrieved LNs during the radical cholecystectomy, even among major HPB centers [16, 17]. Thus, previous studies included patients with less than 6 LNs retrieved when examining the prognostic significance of LNR in GBC, and these results may be biased.

The present study aims to evaluate the prognostic value of LNR in the advanced GBC patients (T2–4 stages) with at least 6 LNs retrieved.

## Materials and methods

### Patients

**Fig. 1:**
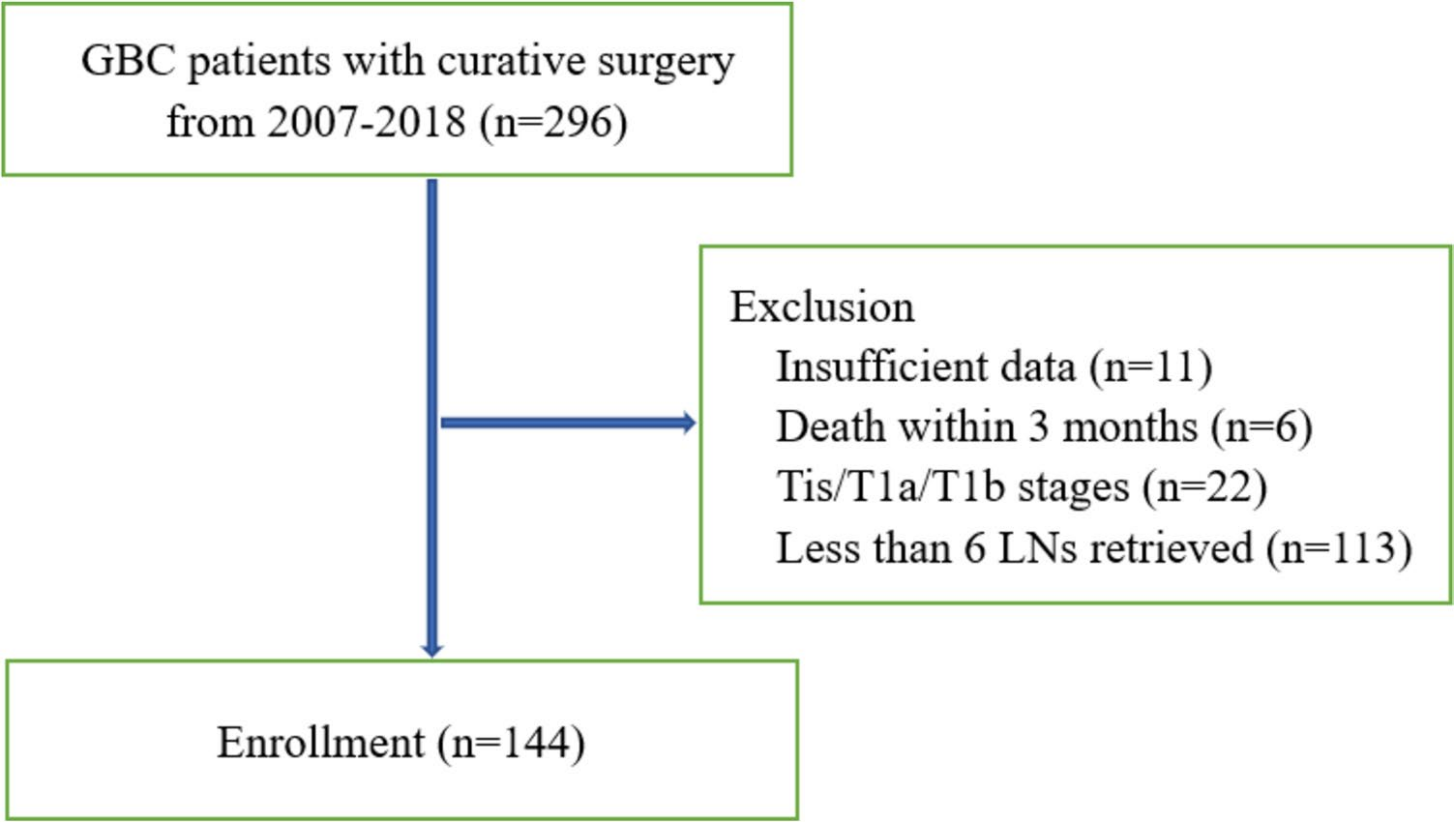
Flow chart showing patients selection

Between January 2007 and January 2018, 296 GBC patients underwent radical cholecystectomy at the Division of Biliary Surgery, West China Hospital of Sichuan University. All the final diagnoses of GBC were confirmed by postoperative pathologic examination. Patients with insufficient clinicopathologic data, death within 3 months after surgery, Tis/T1a/T1b stages, and less than 6 LNs retrieved were excluded (Fig. 1). Ultimately, 144 advanced GBC patients (T2–4 stages) who underwent curative surgery with at least 6 LNs retrieved were included.

### Surgical procedure

All the 144 advanced GBC patients underwent radical cholecystectomy, which involved liver resection and LN dissection. For the incidentally GBC diagnosed after simple cholecystectomy for benign diseases, radical re-resection was performed for curative intent. The surgical procedure varied depends on the extent of the tumor invasion. The extent of liver resection was variable according to the extent of liver infiltration, including wedge resection, segment IVb and V resection, right hemihepatectomy, and right trisegmentectomy. LN dissection was performed in all patients either D1 or D2 dissection, and at least 6 LNs were retrieved. D1 dissection involved LNs around the common hepatic artery and hepatoduodenal ligament, whereas D2 dissection included more LNs around the post-pancreatic duodenum and abdominal arteries. Extrahepatic bile duct resection was performed in the patients with jaundice or positive cystic duct margin. Adjacent organs were also resected for curative intent in cases of certain infiltration, including the stomach, duodenum, and colon. Portal vein and hepatic artery reconstruction were performed if necessary.

Postoperative complications were classified by the Clavien-Dindo grading system, and grade III or higher complications within 3 months after surgery were recorded. R0 resection is defined as complete resection of the tumor with microscopically negative resection margin, while R1 resection is defined as the clean removal of the tumor with microscopically positive margin.

### Patients’ follow-up

All the patients were strictly followed-up retrospectively via outpatient check-ups or telephone interview. Liver functions, tumor markers, and abdominal ultrasound were conducted every 3 months after surgery. Abdominal computed tomography or magnetic resonance imaging was further conducted if tumor recurrence was suspected. The OS was defined as the period from the date of surgery to death or last follow-up, whereas disease-free survival (DFS) was defined as the period from the date of surgery to the date of first recurrence. Patients with LNs metastasis, especially those with more than 3 positive LNs, are potential candidates for adjuvant chemotherapy with gemcitabine-based regimens.

### Statistical analysis

All statistical analyses were based on the SPSS version 25.0 (IBM SPSS, Chicago, IL, USA) and GraphPad prism 9. The quantitative data in the study fits the normal distribution or skew-normal distribution, and were presented as median (range). The qualitative data in the study were presented as number (percentage). Independent samples *t*-test was performed to detect significant differences between two groups of quantitative data, and Fisher’s exact test or chi-square test was performed to detect significant differences between two groups of qualitative data. A receiver operating characteristic (ROC) curve was used to identify the optimal cut-off point for LNR. Univariate analysis of OS was estimated using the Kaplan-Meier log rank test. Factors with *P*<0.05 in the univariate analysis were subjected to multivariate analysis using the Cox proportional-hazards model. The hazard ratios (HR) and corresponding 95% confidence intervals (CI) were calculated during the multivariable analysis. *P* < 0.05 was considered statistically significant.

## Results

### The baseline characteristics of the advanced GBC patients

The baseline characteristics of the advanced GBC patients enrolled in the study are shown in Table 1. Of the 144 patients identified, the median age was 63 years (range from 36 to 85 years). The whole cohort included 95 (65.9%) females and 49 (34.1%) males. The median preoperative total bilirubin (TB), alanine aminotransferase (ALT), and aspartate transaminase (AST) were 11.8 μmol/L (range from 4.7 to 261.6 μmol/L), 24 IU/L (range from 6 to 842 IU/L), and 25 IU/L (range from 13 to 850 IU/L), respectively. The median preoperative CA19-9 and CA125 were 22.2 U/mL (range from 0.1 to >1000 U/mL) and 19.7 U/mL (range from 2.1 to 1567.0 U/mL), respectively.

**Table 1:** Clinicopathological characteristics of the 144 advanced GBC patients

Variables	Number (%) or median (range)
Age (years)	63 (36–85)
Sex (female)	95 (65.9%)
TB (μmol/L)	11.8 (4.7–261.6)
ALT (IU/L)	24 (6–842)
AST (IU/L)	25 (13–850)
ALB (g/L)	41.1 (26.4–52.4)
CA19-9 (U/mL)	22.2 (0.1–>1000)
CA125 (U/mL)	19.7 (2.1–1567.0)
Extent of liver resection	
Wedge resection	18 (12.5%)
Segment IVb and V resection	84 (58.3%)
Right hemihepatectomy	34 (23.6%)
Right trisegmentectomy	8 (5.6%)
Lymph node dissection	
D1	92 (63.9%)
D2	52 (36.1%)
Extrahepatic bile duct resection	76 (52.8%)
Adjacent organs resection	15 (10.4%)
Portal vein reconstruction	13 (9.0%)
Hepatic artery reconstruction	16 (11.1%)
Estimated blood loss (mL)	215 (50–800)
Tumor stage	
T2	62 (43.1%)
T3	57 (39.6%)
T4	25 (17.3%)
LNs metastasis	121 (84.0%)
No. of positive LNs	3 (0–13)
No. of retrieved LNs	8 (6–18)
LNR	0.33 (0–1)
R0 resection	119 (82.6%)
Tumor differentiation	
Poorly	85 (59.0%)
Well, moderately	59 (41.0%)
Perineural invasion	33 (22.9%)
Intravascular invasion	29 (20.1%)
Postoperative complications	48 (33.3%)
Postoperative hospital stay (days)	7 (4–47)
Postoperative chemotherapy	32 (22.2%)

*TB*, total bilirubin; *ALT*, alanine aminotransferase; *AST*, aspartate transaminase; *ALB*, albumin; *LNs*, lymph nodes; *LNR*, lymph node ratio

As for the extent of liver resection, 18 (12.5%), 84 (58.3%), 34 (23.6%), and 8 (5.6%) patients underwent wedge resection, segment IVb and V resection, right hemihepatectomy, and right trisegmentectomy, respectively. D1 LN dissection was performed in 92 (63.9%) patients, whereas D2 LN dissection in 52 (36.1%) patients. Extrahepatic bile duct was resected in 76 (52.8%) patients. Adjacent organs were resected in 15 (10.4%) patients, including the stomach and duodenum (*n*=6), and the colon (*n*=9). Portal vein and hepatic artery were reconstructed in 13 (9.0%) and 16 (11.1%) patients, respectively. The median estimated blood loss was 215 mL (range from 50 to 800 mL).

Pathological results revealed that 62 (43.1%), 57 (39.6%), and 25 (17.3%) patients were at T2, T3, and T4 stages, respectively. One hundred and twenty-one (84.0%) patients were found to have LN metastases. The median number of positive LNs and retrieved LNs was 3 (range from 0 to 13) and 8 (range from 6 to 18), respectively. After calculation, the median LNR was 0.33 (range from 0 to 1). Of the 144 patients, 119 (82.6%) achieved an R0 resection. As for the tumor differentiation, 85 (59.0%) patients were poorly differentiated, whereas 59 (41.0%) patients were moderately or well differentiated. Perineural and intravascular invasions were present in 33 (22.9%) and 29 (20.1%) patients, respectively.

Besides, 48 (33.3%) patients suffered postoperative complications, including bile leakage (*n*=26), hepatic failure (*n*=7), lung infection (*n*=5), hemorrhage (*n*=4), peritoneal cavity infection (*n*=4), and pancreatic fistula (*n*=2). The median postoperative hospital stay was 7 days (range from 4 to 47 days). Thirty-two (22.2%) patients received postoperative adjuvant chemotherapy with gemcitabine-based regimens.

### The optimal cut-off point for LNR

**Fig. 2:**
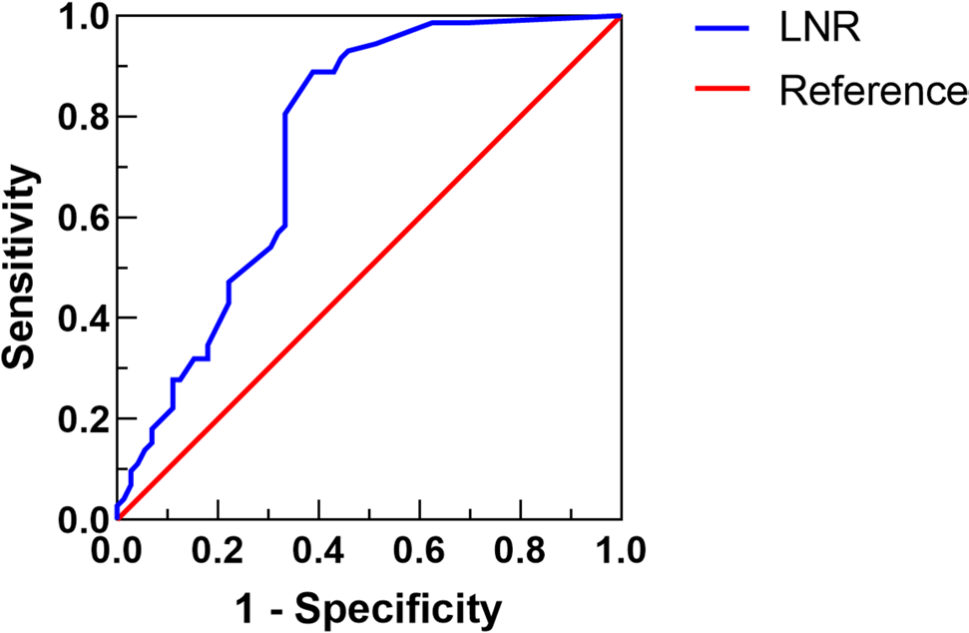
Optimal cut-off point for the LNR was identified by ROC curve analysis

A ROC curve was constructed to determine the optimal cut-off point for LNR (Fig. 2). The sensitivity and specificity of LNR in OS were 83.3% and 61.1% (AUC=0.752, 95% CI 0.731–0.902, *P*<0.001), respectively. The optimal cut-off point for LNR was 0.28. The study population was then subdivided into two groups: LNR≤0.28 (56 patients, 38.9%) and LNR>0.28 (88 patients, 61.1%). The clinicopathological characteristics stratified by LNR were analyzed (Table 2). LNR>0.28 was associated with higher rate of D2 LN dissection (*P*=0.004) and higher tumor stages (*P*<0.001). There were no obvious differences in other clinicopathologic parameters with regard to LNR levels.

**Table 2:** Comparison of clinicopathological characteristics of advanced GBC patients with different LNR levels

Variables	LNR≤0.28 (*n*=56)	LNR>0.28 (*n*=88)	*P* value
Age (years)	63 (37–85)	62 (36–80)	0.630
Sex (female)	40 (71.4%)	55 (62.5%)	0.270
TB (μmol/L)	11.9 (5.8–261.6)	11.8 (4.7–236.7)	0.598
ALT (IU/L)	27 (6–172)	23 (9–842)	0.231
AST (IU/L)	24 (13–197)	25 (14–850)	0.248
ALB (g/L)	40.6 (31.9–47.2)	41.3 (26.4–52.4)	0.384
CA19-9 (U/mL)	17.9 (0.1–>1000)	28.4 (0.1–>1000)	0.292
CA125 (U/mL)	17.3 (2.5–367.3)	25.4 (2.1–1657.0)	0.181
Extent of liver resection			0.058
Wedge resection	10 (17.8%)	8 (9.1%)	
Segment IVb and V resection	36 (64.3%)	48 (54.5%)	
Right hemihepatectomy	7 (12.5%)	27 (30.7%)	
Right trisegmentectomy	3 (5.4%)	5 (5.7%)	
Extrahepatic bile duct resection	26 (46.4%)	50 (56.8%)	0.223
Adjacent organs resection	5 (8.9%)	10 (11.3%)	0.782
Portal vein reconstruction	6 (10.7%)	7 (7.9%)	0.562
Hepatic artery reconstruction	6 (10.7%)	10 (11.3%)	0.903
LN dissection			**0.004**
D1	44 (78.6%)	48 (54.5%)	
D2	12 (21.4%)	40 (45.5%)	
Estimated blood loss (mL)	200 (100–550)	250 (50–800)	0.151
Tumor stage			**<0.001**
T2	38 (67.9%)	24 (27.3%)	
T3	13 (23.2%)	44 (50.0%)	
T4	5 (8.9%)	20 (22.7%)	
R0 resection	50 (89.3%)	69 (78.4%)	0.115
Tumor differentiation			0.288
Poorly	30 (53.6%)	55 (62.5%)	
Well, moderately	26 (46.4%)	33 (37.5%)	
Perineural invasion	9 (16.1%)	24 (27.3%)	0.119
Intravascular invasion	7 (12.5%)	22 (25.0%)	0.068
Postoperative complications	16 (28.6%)	32 (36.4%)	0.368
Postoperative stay (days)	7 (4–44)	7 (4–47)	0.173
Postoperative chemotherapy	14 (25.0%)	18 (20.4%)	0.542

*TB* total bilirubin, *ALT* alanine aminotransferase, *AST* aspartate transaminase, *ALB* albumin, *LNs* lymph nodes, *LNR* lymph node ratio

### The survival outcomes

**Fig. 3:**
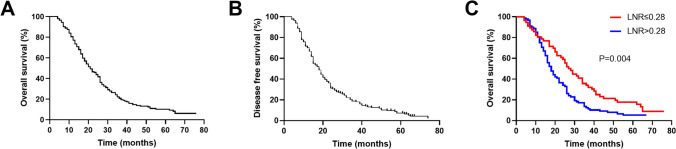
Kaplan-Meier survival curves of patients with advanced GBC. **A** The OS curve of entire 144 patients; **B** The DFS curve of entire 144 patients; **C** OS comparison between patients with LNR≤0.28 and LNR>0.28

Of the 144 advanced GBC patients, 133 (92.4%) patients died before the last follow-up, and 137 (95.1%) patients have the tumor recurrence. The median OS of the entire cohort was 21 months (range from 4 to 76 months). The 1-, 3-, and 5-year OS rates were estimated as 77.1%, 20.8%, and 10.3%, respectively (Fig. 3A). The median DFS of the entire cohort was 18 months (range from 4 to 74 months). The 1-, 3-, and 5-year DFS rates were estimated as 70.1%, 18.8%, and 7.2%, respectively (Fig. 3B). In total, patients with LNR≤0.28 had a significantly longer median OS than those with LNR>0.28 (27.5 vs 18 months, *P*=0.004, Fig. 3C).

**Fig. 4:**
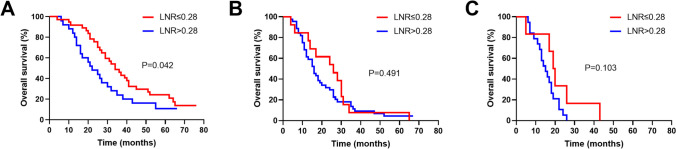
Subgroups of Kaplan-Meier OS curves between patients with LNR≤0.28 and LNR>0.28. **A** T2 stage; **B** T3 stage; **C** T4 stage

Subgroup analyses were performed according to the tumor stage and the extent of LN dissection, which had revealed significant correlation with LNR. As for the tumor stage, patients with LNR≤0.28 had a significantly longer median OS than those with LNR>0.28 at T2 stage (34 vs 22 months, *P*=0.042, Fig. 4A). However, no survival differences were observed between patients with LNR≤0.28 and LNR>0.28 at T3 (26 vs 16 months, *P*=0.491, Fig. 4B) and T4 (19.5 vs 15 months, *P*=0.103, Fig. 4C) stages.

**Fig. 5:**
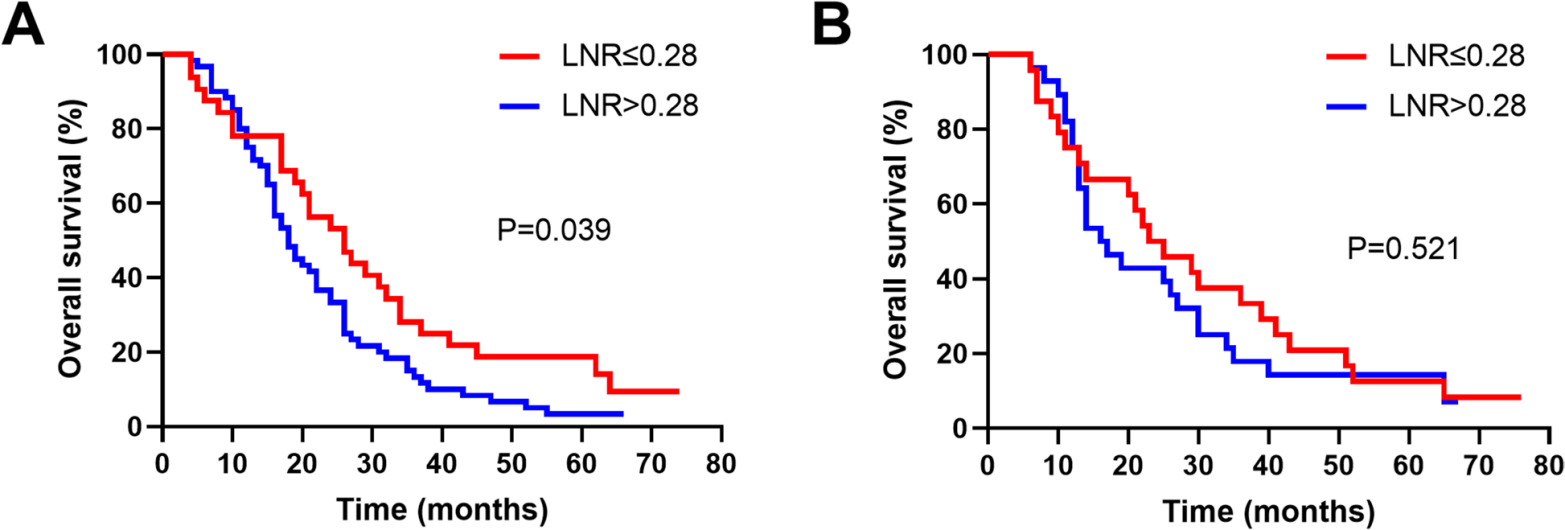
Subgroups of Kaplan-Meier OS curves between patients with LNR≤0.28 and LNR>0.28. **A** D1 LN dissection; **B** D2 LN dissection

As for the extent of LN dissection, patients with LNR≤0.28 had a significantly longer median OS than those with LNR>0.28 while D1 dissection (26 vs 18 months, *P*=0.039, Fig. 5A). However, no survival difference was observed between patients with LNR≤0.28 and LNR>0.28 while D2 dissection (24 vs 16.5 months, *P*=0.521, Fig. 5B).

### Prognostic factors for advanced GBC

To identify the independent prognostic factors for advanced GBC, various clinicopathologic parameters were analyzed by univariate and multivariate Cox proportional hazards regression models. The univariate analysis results revealed that extent of liver resection (*P*=0.001), extrahepatic bile duct resection (*P*=0.026), tumor stage (*P*<0.001), LNR (*P*=0.004), margin status (*P*=0.009), tumor differentiation (*P*=0.003), and perineural invasion (*P*=0.041) were significantly associated with the OS (Table 3). Significantly, patients with ≤3 positive LNs had longer median OS than those with >3 positive LNs; however, it did not reach statistical significance (25 vs 18 months, *P*=0.081). The above clinicopathologic parameters with *P* < 0.05 in univariate analysis were included in multivariable Cox regression model. The results showed that tumor stage (T2 vs T3/T4; *P*=0.002, HR 1.596; 95% CI 1.195–2.132), LNR (≤0.28 vs >0.28; *P*=0.029, HR 0.666; 95% CI 0.463–0.958), margin status (R0 vs R1; *P*=0.011, HR 1.828; 95% CI 1.148–2.910), and tumor differentiation (poorly vs well/moderately; *P*=0.048, HR 0.670; 95% CI 0.589–0.892) were independent prognostic factors for OS.

**Table 3:** Univariate and multivariable analysis of advanced GBC associated with OS

Variables	Univariate analysis	Multivariate analysis		
	*P*	HR	95% CI	*P*
Age (≤62 vs >62 years)	0.196			
Sex (males vs females)	0.829			
TB (≤11.8 vs >11.8 μmol/L)	0.825			
ALT (≤26 vs >26 IU/L)	0.329			
AST (≤25 vs >25 IU/L)	0.620			
ALB (≤41.6 vs >41.6 g/L)	0.225			
CA19-9 (≤23.1 vs >23.1 U/mL)	0.847			
CA125 (≤19.7 vs >19.7 U/mL)	0.354			
Extent of liver resection (major vs minor hepatectomy)*	**0.001**	1.166	0.755–1.800	0.489
LN dissection (D1 vs D2 dissection)	0.339			
Extrahepatic bile duct resection (yes vs no)	**0.026**	1.490	0.698–3.180	0.303
Adjacent organs resection (yes vs no)	0.477			
Portal vein reconstruction (yes vs no)	0.129			
Hepatic artery reconstruction (yes vs no)	0.067			
Estimated blood loss (≤220 vs >220 mL)	0.050			
Tumor stage (T2 vs T3/T4)	**<0.001**	**1.596**	**1.195–2.132**	**0.002**
LNR (≤0.28 vs >0.28)	**0.004**	**0.666**	**0.463–0.958**	**0.029**
Number of LN metastasis (≤3 vs >3)	0.081			
Margin status (R0 vs R1)	**0.009**	**1.828**	**1.148–2.910**	**0.011**
Tumor differentiation (poorly vs well/moderately)	**0.003**	**0.670**	**0.589–0.892**	**0.048**
Perineural invasion (yes vs no)	**0.041**	1.044	0.669–1.629	0.849
Intravascular invasion (yes vs no)	0.824			
Postoperative complications (yes vs no)	0.830			
Postoperative stay (≤7 vs >7 days)	0.903			
Postoperative chemotherapy (yes vs no)	0.454			

*Major hepatectomy included right hemihepatectomy and right trisegmentectomy; minor hepatectomy included wedge resection and segment *IVb* and *V* resection. *TB* total bilirubin, *ALT* alanine aminotransferase, *AST* aspartate transaminase, *ALB* albumin, *LNs* lymph nodes, *LNR* lymph node ratio

### The prognostic significance of postoperative chemotherapy based on LNR

**Fig. 6:**
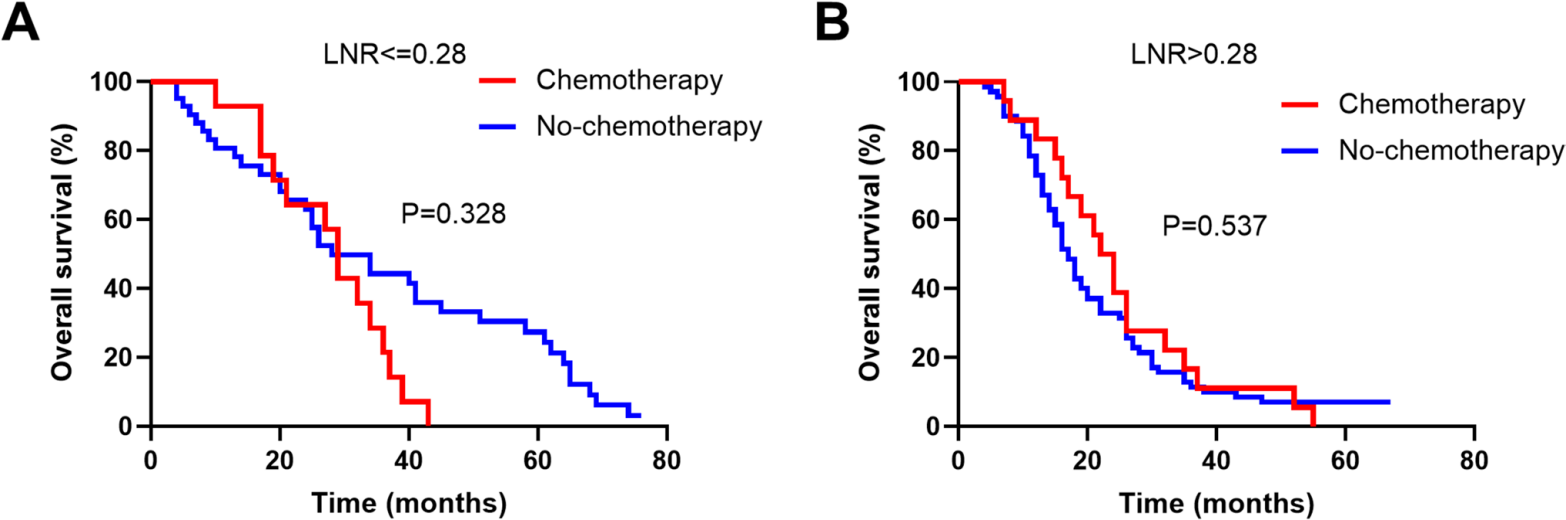
Kaplan-Meier OS curves between patients with or without postoperative chemotherapy. **A** LNR≤0.28; **B** LNR>0.28

Postoperative chemotherapy is crucial in the multimodality therapy of advanced GBC patients. Thus, we further explored the prognostic significance of postoperative chemotherapy based on different LNR level. There was no significant difference in OS between patients who received chemotherapy and those who did not in case of LNR ≤0.28 (Fig. 6A) or LNR>0.28 (Fig. 6B). However, patients with postoperative chemotherapy have longer median OS than without chemotherapy whether LNR≤0.28 (29 vs 28 months) or LNR>0.28 (23 vs 17 months). Although not statistically significant, the improved median OS are significant for patients with advanced tumors. Thus, we recommended that postoperative chemotherapy should be performed in advanced GBC patients, especially in patients with LNR>0.28.

## Discussion

Although relatively unusual, GBC is the most common type of biliary tract carcinomas. GBC lacks submucosa and has an extremely thin proper muscle layer, which increases the propensity for local invasion and distant metastases [18, 19]. Thus, GBC is a devastating malignant neoplasm with an extremely poor prognosis. Radical resection remains the best treatment option; however, most patients are diagnosed at advanced stages. Besides, GBC has the dilemma of lack of effective prognostic markers, and thus we evaluated the prognostic significance of LNR in GBC. The main findings in our study were as follows: (1) LNR rather than the number of metastatic LNs was correlated to advanced GBC prognosis and was a potential prognostic factor for advanced GBC with at least 6 LNs retrieved; (2) The optimal cut-off point for LNR was 0.28, according to the ROC curve; (3) Multivariate analysis showed that tumor stage, LNR, margin status, and tumor differentiation were independent prognostic factors for OS.

LN metastasis is one of the most important prognostic factors for GBC after curative resection. Many issues about LN metastasis in GBC still need clarification. Firstly, the optimal extent of LN dissection is very well established in some gastrointestinal malignancies such as gastric and rectal cancer [20, 21], but not yet in GBC. Some scholars recommended that D2 LN dissection should be performed in all patients for the intent of better staging [6], while others recommended that extensive lymphadenectomy should be strictly limited owing to serious morbidity and mortality [22]. Even so, most investigators have approved that LN dissection with at least 6 retrieved provides more accurate pathologic staging for GBC [17, 23, 24]. Thus, the 8th edition of the AJCC tumor staging system for GBC recommended that at least 6 LNs should be examined. In our study, among the patients with at least 6 LNs retrieved, 63.9% of the cases performed D1 LN dissection and the remaining D2 LN dissection. Hamad A et al. reported that LN metastases were found in 41.7% GBC patients with T1b-4 stages [25]. In addition, other scholars also reported that LN metastases rate varied by tumor stages, and up to 45–80% in T2–4 stages [26]. In our study, the incidence of LN metastases in advanced GBC with T2–4 stages was 84.0%. The incidence of LN metastases in our study was higher than those reported by other investigators owing to higher LNs retrieved. These results indicated that the rate of positive LNs maybe improved with more LNs retrieved.

The prognostic impact of LNs status in GBC patients has been investigated by many researchers. Generally, GBC patients with LN metastasis have worse prognosis compared to those without LN metastases [27]. Thus, the early LN staging is based on the presence or absence of LN metastases. According to the 7th edition of AJCC classification, LN metastases were divided into N1 and N2 levels based on their anatomical location. However, recent studies have revealed that the number of positive LNs is associated with GBC prognosis [28]. Thus, the latest version of LN staging was based on the number of positive LNs rather than the location. In our study, patients with no more than 4 positive LNs had better prognosis than those with at least 4 positive LNs, however, it did not reach statistical significance (*P*=0.081). Thus, other LN prognostic schemes such as LNR should be explored.

The prognostic impact of LNR has been reported in GBC patients by some investigators and remains controversial. Negi SS et al. found that LNR was a strong prognostic factor after curative resection for GBC, and the optimal LNR cut-off point was determined to be 0.50 [15]. Birnbaum DJ et al. also found that LNR, the cut-off point of 0.15, stratified the prognoses of GBC patients, but not the site of metastatic LNs [6]. Furthermore, Amini N et al. indicated that LNR performed better than the AJCC LN staging system, especially for patients with four or more LNs examined [29]. However, Chen C et al. conducted a multi-institutional study to evaluate the prognosis including metastatic LNs, log odds of metastatic LNs, and LNR, and concluded that number of metastatic LNs was the optimal LN staging system in evaluating GBC prognosis [30]. Significantly, the above studies included patients with less than 6 LNs retrieved when examining the prognostic significance of LNR, and the results may be biased. In our study, a higher LNR was associated with poorer OS by univariate analysis, and the optimal LNR cut-off point was 0.28. Multivariate analysis further identified that the LNR was an independent prognostic factor for advanced GBC. Besides, subgroup analyses were performed according to the tumor stage and the extent of LN dissection, which was correlated with the LNR. The results indicated that higher LNR predict poor prognosis for advanced GBC with T2 stage or D1 LN dissection. However, we did not observe the prognostic significance of LNR in GBC patients with T3–4 stages or D2 LN dissection. The possible reasons are as follows: patients with T3–4 stages or D2 LN dissection involved with surrounding organs invasion or more extensive LN metastases, and thus, the LNR alone cannot accurately predict these extremely advanced patients. Next, we will investigate the combination of LNR and other prognostic factors to predict these patients.

Our study also showed that tumor stage, margin status, and tumor differentiation were independent prognostic factors for advanced GBC. These prognostic factors in GBC have been proved in some studies [31, 32], which emphasized the importance of early diagnosis, curative resection, and negative margin for GBC patients. The limitation of our study should be considered. Our study was performed in a retrospective design with a single-center sample size. Further multi-center, larger prospective trials are required to verify the reliability of our results.

## Conclusion

LNR is associated with advanced GBC prognosis and has advantages in providing a more precise prognostic evaluation for advanced GBC with at least 6 LNs retrieved. However, there is a need to ensure a high-quality lymphadenectomy and subsequent pathological examination of all resected LNs in resected GBC patients.

## Data Availability

The data generated and analyzed during the present study can be obtained from the corresponding author upon reasonable request.
